# Phenotypic characterization of X-linked hypophosphatemia in pediatric Spanish population

**DOI:** 10.1186/s13023-021-01729-0

**Published:** 2021-02-27

**Authors:** Enrique Rodríguez-Rubio, Helena Gil-Peña, Sara Chocron, Leire Madariaga, Francisco de la Cerda-Ojeda, Marta Fernández-Fernández, Carmen de Lucas-Collantes, Marta Gil, María Isabel Luis-Yanes, Inés Vergara, Juan David González-Rodríguez, Susana Ferrando, Montserrat Antón-Gamero, Marta Carrasco Hidalgo-Barquero, Angustias Fernández-Escribano, Mº Ángeles Fernández-Maseda, Laura Espinosa, Aniana Oliet, Antonio Vicente, Gema Ariceta, Fernando Santos, RenalTubeGroup

**Affiliations:** 1 Pediatric Research, Medicine Department, University of Oviedo, Oviedo, Spain; 2 AGC Pediatría, Hospital Universitario Central de Asturias, Oviedo, Spain; 3 Servicio de Nefrología Pediátrica, Hospital Vall D’Hebron, Universitat Autónoma de Barcelona, Barcelona, Spain; 4 Servicio Nefrología Pediátrica, IIS Biocruces-Bizkaia, Universidad del País Vasco UPV/EHU, Hospital Universitario Cruces, Barakaldo, Spain; 5 Unidad de Nefrología Pediátrica, Hospital Virgen del Rocío, Sevilla, Spain; 6 Servicio Pediatría, Complejo Asistencial Universitario de León, León, Spain; 7 Servicio Nefrología, Hospital Niño Jesús, Madrid, Spain; 8 Servicio Pediatría, Hospital Universitario de Santiago de Compostela, Santiago de Compostela, Spain; 9 Servicio Pediatría, Hospital Universitario Nuestra Señora de Candelaria, Santa Cruz de Tenerife, Spain; 10 Servicio Pediatría, Complexo Hospitalario Universitario A Coruña (CHUAC), A Coruña, Spain; 11 Unidad de Nefrología, Hospital General Universitario Santa Lucia, Cartagena, Spain; 12 Servicio de Pediatría, Hospital Clínico Universitario de Valencia, Valencia, Spain; 13 Unidad Nefrología Pediátrica, Hospital Universitario Reina Sofia, Córdoba, Spain; 14 Unidad de Nefrología Pediátrica, Hospital Universitario de Badajoz, Badajoz, Spain; 15 ServicioNefrología Infantil, Hospital Infantil Gregorio Marañón, Madrid, Spain; 16 Unidad de Nefrología Pediátrica, Hospital Virgen de la Salud, Toledo, Spain; 17 Servicio Nefrología infantile, Hospital Universitario Infantil La Paz, Madrid, Spain; 18 Servicio Nefrología, Hospital Severo Ochoa, Leganés, Spain; 19 Servicio Pediatría, Hospital Vega Baja, Orihuela, Spain

**Keywords:** XLH, Inherited hypophosphatemia, Growth retardation, Bone deformities, Rickets

## Abstract

**Background:**

X-linked hypophosphatemia (XLH) is a hereditary rare disease caused by loss-of-function mutations in *PHEX* gene leading tohypophosphatemia and high renal loss of phosphate. Rickets and growth retardation are the major manifestations of XLH in children, but there is a broad phenotypic variability. Few publications have reported large series of patients. Current data on the clinical spectrum of the disease, the correlation with the underlying gene mutations, and the long-term outcome of patients on conventional treatment are needed, particularly because of the recent availability of new specific medications to treat XLH.

**Results:**

The RenalTube database was used to retrospectively analyze 48 Spanish patients (15 men) from 39 different families, ranging from 3 months to 8 years and 2 months of age at the time of diagnosis (median age of 2.0 years), and with XLH confirmed by genetic analysis. Bone deformities, radiological signs of active rickets and growth retardation were the most common findings at diagnosis. Mean (± SEM) height was − 1.89 ± 0.19 SDS and 55% (22/40) of patients had height SDS below—2. All cases had hypophosphatemia, serum phosphate being − 2.81 ± 0.11 SDS. Clinical manifestations and severity of the disease were similar in both genders. No genotype—phenotype correlation was found. Conventional treatment did not attenuate growth retardation after a median follow up of 7.42 years (IQR = 11.26; n = 26 patients) and failed to normalize serum concentrations of phosphate. Eleven patients had mild hyperparathyroidism and 8 patients nephrocalcinosis.

**Conclusions:**

This study shows that growth retardation and rickets were the most prevalent clinical manifestations at diagnosis in a large series of Spanish pediatric patients with XLH confirmed by mutations in the *PHEX* gene. Traditional treatment with phosphate and vitamin D supplements did not improve height or corrected hypophosphatemia and was associated with a risk of hyperparathyroidism and nephrocalcinosis. The severity of the disease was similar in males and females.

## Background

X-linked hypophosphatemic rickets (XLH) (OMIM 307800) (ORPHA 89936) is the most common hereditary rickets [1–5] with an estimated prevalence of 1:20,000 [6, 7]. It follows an X-linked dominant transmission [8]. The disease is caused by a defective function of *PHEX* gene [1, 9–13], leading to elevated circulating concentrations of fibroblast growth factor 23 (FGF23) [14], relatively low levels of 1,25 dihydroxyvitamin d [1,25(OH)_2_D], hyperphosphaturia secondary to decreased proximal tubular reabsorption of phosphate and hypophosphatemia [8, 10, 15]. Classical, conventional treatment of XLH is based on the administration of phosphate supplements and 1-alpha hydroxylated derivates of vitamin D [16]. The wider availability of genetic studies and the recent development of an anti-FGF23 antibody, burosumab, as novel and promising therapy [10, 17] have resulted in a growing current interest for XLH.

We here report the clinical manifestations at diagnosis and follow-up of a large series of Spanish patients included in the online database RenalTube [18]. This study is justified at least by the following reasons: (1) XLH is a rare disease and few publications provide data on large series of patients; (2) XLH has a broad phenotypic variability and additional information is required to better characterize the clinical spectrum of the disease and to explain why the number of cases diagnosed usually does not correspond to the estimated prevalence of the disease; (3) It is important to share data of patients with genetically confirmed XLH in order to facilitate the finding of a potential phenotype—genotype correlation and to have current data that can be compared for the assessment of the new therapies.

## Results

**Fig. 1:**
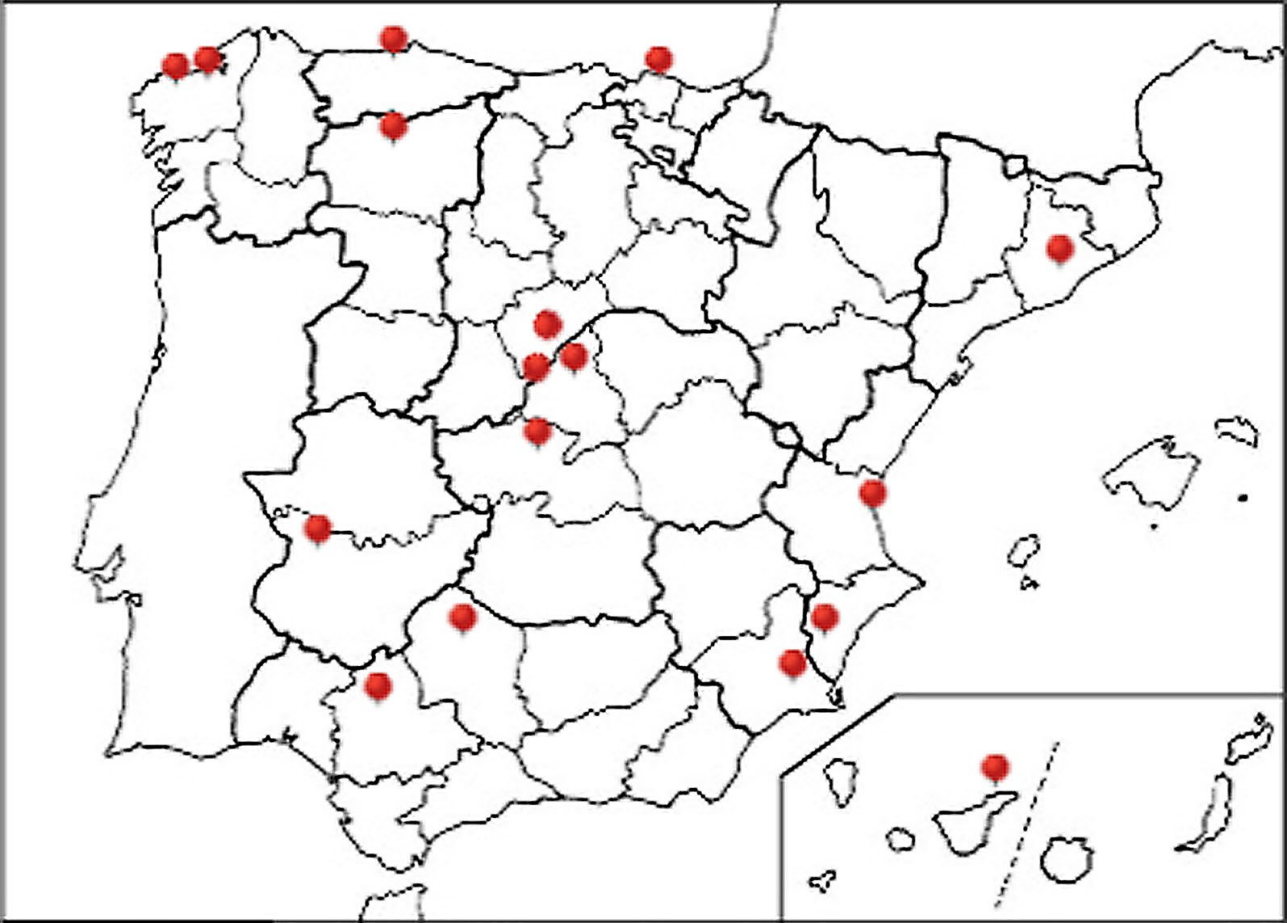
Geographical distribution of the Spanish hospitals participating in the study

Forty-eight patients included in the RenalTube database with the diagnosis of XLH confirmed by defect-of-function variants found in the *PHEX* gene were analyzed. All variants had been identified as pathogenic. Sixteen patients (33%) had variants with strong evidence of pathogenicity (nonsense, frameshift, deletions) while the other 32 (67%) harbored variants with very strong evidence of pathogenicity (SNPs). Demographic and genetic data from patients are shown in Table 1. Patients were from 39 families and were being followed in pediatric nephrology units of 17 Spanish hospitals (Fig. 1). Fifteen patients were males and 33 females. Median age at diagnosis was 2.0 (IQR 2.6) years and the age ranged from 3 months to 8 years 2 months.

**Table 1:** Demographic and genetic data of 48 patients belonging to 39 families (Roman number indicates family)

Patient	Relationship	Sex	cDNA mutation	Protein mutation	Variant type
I.1	Index	F	c.758_759delTT	p.F26Cfs	P (PVS1)
I.2	Sister	F	c.758_759delTT	p.F26Cfs	P (PVS1)
II.1	Index	F	c.2223_2224delAC	p.A514Afs516X	P (PVS1)
III.1	Index	F	c.1578_1579delAA	p.K299Nfs304X	P (PVS1)
IV.1	Index	M	c.893A>T	p.N71I	P (PS1)
IV.2	Brother	M	c.893A>T	p.N71I	P (PS1)
V.1	Index	F	c.2633G>C	p.R651P	P (PS1)
VI.1	Index	F	c.1885C>T	p.Q402X	P (PVS1)
VII.1	Index	M	c.?-2664dup2949-?	Splice region variant	P (PVS1)
VII.2	Brother	M	c.?-2664dup2949-?	Splice region variant	P (PVS1)
VII.3	Daughter	F	c.?-2664dup2949-?	Splice region variant	P (PVS1)
VII.4	Daughter	F	c.?-2664dup2949-?	Splice region variant	P (PVS1)
VIII.1	Index	F	c.886insT	p.K69X	P (PVS1)
IX.1	Index	F	c.2048G>A	p.W456X	P (PVS1)
X.1	Index	F	g.22099152G>T	Splice region variant	P (PVS1)
XI.1	Index	M	c.2648_?del	p.A656_?del	P (PVS1)
XII.1	Index	F	c.2327_?del	p.R549_?del	P (PVS1)
XIII.1	Index	F	g.22168393_delA	Splice region variant	P (PVS1)
XIV.1	Index	F	c.1552C>T	p.R291X	P (PVS1)
XV.1	Index	F	g.22190503G>A	Splice region variant	P (PVS1)
XVI.1	Index	F	c.889_893delGTAAA	p.V70Sfs77X	P (PVS1)
XVII.1	Index	F	c.2086_?del	p.A469_?del	P (PVS1)
XVIII.1	Index	F	c.1180T>C	p.W167R	P (PS1)
XIX.1	Index	F	c.2282C>T	p.P534L	P (PS1)
XX.1	Index	F	c.2416G>A	p.G579R	P (PS1)
XXI.1	Index	F	c.2920C>T	p.R747X	P (PVS1)
XXII.1	Index	F	c.2920C>T	p.R747X	P (PVS1)
XXIII.1	Index	M	c.1152delA	p.L157Lfs220X	P (PVS1)
XXIV.1	Index	F	c.1572C>A,c.1580_1582delTGA	p.Y297X	P (PVS1)
XXV.1	Index	F	g.22076478A>T	Splice region variant	P (PVS1)
XXVI.1	Index	F	g.22190507G>A	Splice region variant	P (PVS1)
XXVII.1	Index	F	c.889_893delGTAAA	p.V70Sfs77X	P (PVS1)
XXVII.2	Father	M	c.889_893delGTAAA	p.V70Sfs77X	P (PVS1)
XXVIII.1	Index	M	c.2879G>T	p.C733F	P (PS1)
XXVIII.2	Sister	F	c.2879G>T	p.C733F	P (PS1)
XXIX.1	Index	F	c.2642T>C	p.F654S	P (PS1)
XXX.1	Index	M	c.2387T>G	p.L569R	P (PS1)
XXX.2	Mother	F	c.2387T>G	p.L569R	P (PS1)
XXXI.1	Index	F	c.2085G>C	p.K468N	P (PS1)
XXXII.1	Index	M	c.2282C>T	p.P534L	P (PS1)
XXXIII.1	Index	M	g.22033125T>G	Splice region variant	P (PVS1)
XXXIV.1	Index	M	c.2380C>T	p.R567X	P (PVS1)
XXXV.1	Index	M	c.2005G>T	p.V442P	P (PS1)
XXXV.2	Mother	F	c.2005G>T	p.V442P	P (PS1)
XXXVI.1	Index	F	c.2416G>A	G579R	P (PS1)
XXXVII.1	Index	M	g.22099152G>A	Splice region variant	P (PVS1)
XXXVIII.1	Index	F	c.2617delG	p.D646Ifs	P (PVS1)
XXXIX.1	Index	M	c.1363_1364delTC	p.S228Pfs236X	P (PVS1)

F: female, M: male

P: pathogenic

PVS1: very strong evidence of pathogenicity according to reference 19

PS1: strong evidence of pathogenicity according to reference 19

Presenting manifestations are shown in Table 2 for each patient. Bone deformities and radiological signs of active rickets were the most frequent findings leading to diagnosis. Ten patients were diagnosed because of family screening. Age at diagnosis of these patients was no different from that of the rest of the series as it ranged from 0.5 to 8 years with a median age of 1.04 years.

**Table 2:** Clinical manifestations at diagnosis

Patient	Age at diagnosis	Bone deformities	Active rickets	Longitudinal growth retardation (≤ 2 SDS)	Dental problems
I.1	3 m	No	–	No	–
I.2	1 y	Yes	–	Yes	–
II.1	1 y 5 m	Yes	Yes	Yes	No
III.1	1 y 6 m	–	Yes	Yes	Yes
IV.1	1 y 4 m	No	No	Yes	Yes
IV.2	4 y	No	No	No	No
V.1	5 y	Yes	Yes	Yes	No
VI.1	2 y	Yes	No	No	No
VII.1	8 y	–	–	–	–
VII.2	2 y	–	Yes	–	No
VII.3	11 m	–	Yes	Yes	No
VII.4	1 y	–	–	Yes	–
VIII.1	4 y	Yes	Yes	No	No
IX.1	5 y	Yes	Yes	No	No
X.1	5 y	Yes	Yes	Yes	No
XI.1	2 y 3 m	Yes	Yes	Yes	No
XII.1	2 y	Yes	Yes	–	–
XIII.1	5 y	–	Yes	–	–
XIV.1	9 m	Yes	Yes	No	No
XV.1	1 y 1 m	Yes	Yes	Yes	–
XVI.1	4 y	Yes	–	Yes	No
XVII.1	5 y	–	–	Yes	–
XVIII.1	2 y	Yes	Yes	Yes	No
XIX.1	7 m	Yes	Yes	–	No
XX.1	2 y 1 m	Yes	–	Yes	–
XXI.1	4 y 5 m	Yes	Yes	No	No
XXII.1	4 y	Yes	Yes	Yes	No
XIII.1	2 y	Yes	Yes	No	No
XXIV.1	3 y	Yes	–	No	–
XXV.1	1 y 6	Yes	Yes	No	No
XXVI.1	4 y	Yes	Yes	Yes	No
XXVII.1	6 m	Yes	Yes	No	No
XXVII.2	8 y	–	Yes	–	No
XXVIII.1	1 y 6 m	Yes	–	No	–
XXVIII.2	6 m	Yes	Yes	No	No
XXIX.1	1 y 9 m	Yes	Yes	Yes	No
XXX.1	1 y 1 m	Yes	Yes	Yes	–
XXX.2	1 y 6 m	–	Yes	–	No
XXXI.1	8 y2 m	Yes	Yes	Yes	No
XXXII.1	1 y 6 m	Yes	Yes	No	No
XXXIII.1	5 y	–	–	Yes	–
XXXIV.1	2 y 2 m	Yes	Yes	No	No
XXXV.1	7 m	Yes	Yes	No	–
XXXV.2	2 y 6 m	Yes	Yes	Yes	–
XXXVI.1	6 m	Yes	Yes	No	No
XXXVII.1	1 y 9 m	Yes	Yes	Yes	Yes
XXXVIII.1	1 y 6 m	Yes	Yes	No	No
XXXIX.1	2 y 2 m	Yes	Yes	–	No
Percentage of patients		P/A/U	P/A/U	P/A/U	P/A/U
		73/6/21	73/6/21	46/38/17	6/63/31

m: month, y: year

SDS: standard deviation score. Dash: information in this field was missing from the database

P/A/U: present/absent/unreported

**Fig. 2:**
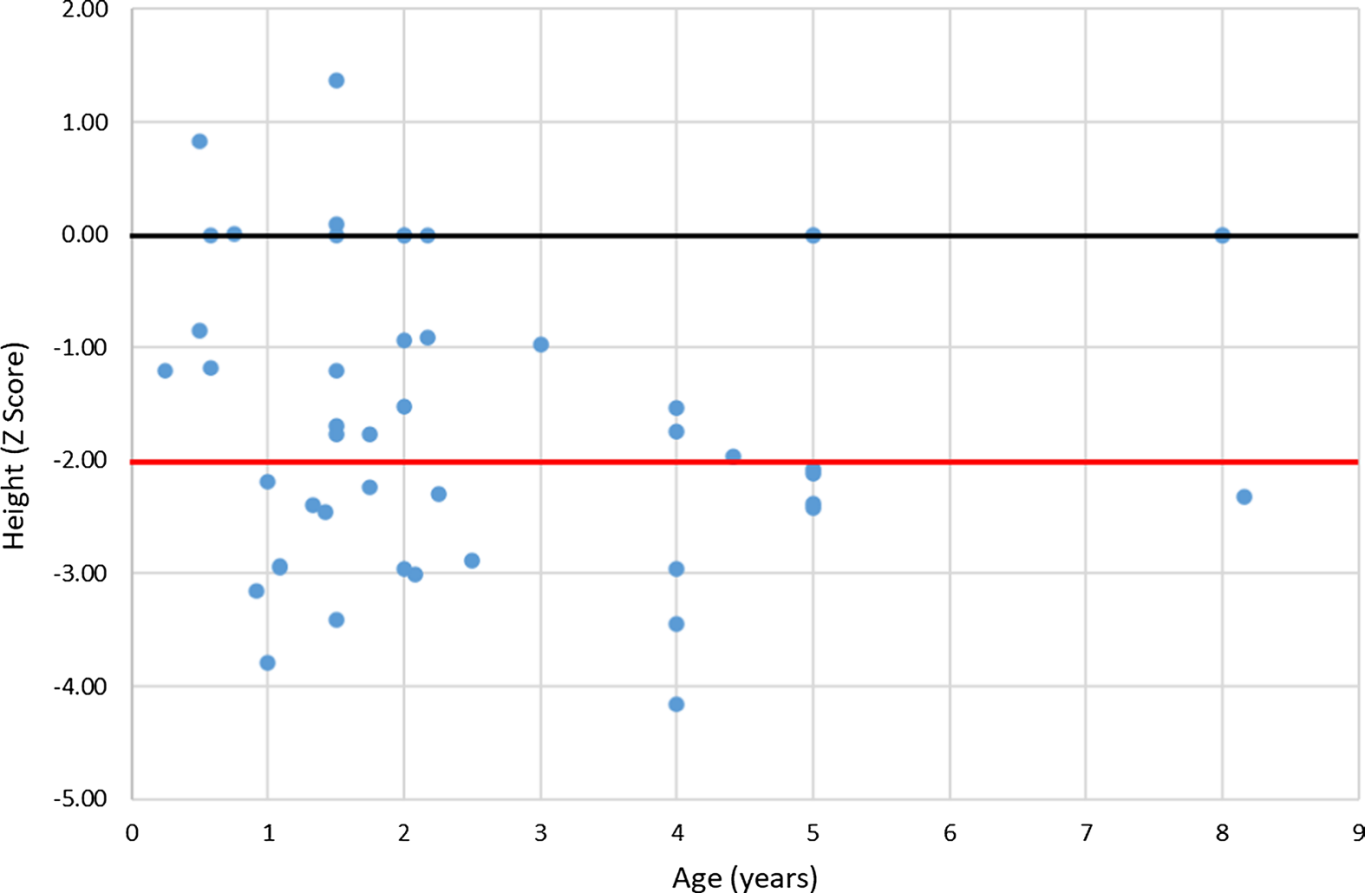
Height at diagnosis (n = 40). Black line: 0 SD; red line: − 2 SD

Twenty-two out of40 patients (55%) in whom the height was registered presented growth retardation (height ≤ 2 SDS). Patients’ height (X ± SEM) was − 1.89 ± 0.19 SDS (n = 40) (Fig. 2). In 87% (35/40) the height was below the 50th percentile. Weight was − 0.88 ± 0.14 SDS (n = 41) and body mass index 0.2 ± 0.15 SDS (n = 40).

Biochemical findings at diagnosis are shown in Table 3. Mean values (± SEM) of available data were serum phosphate 2.7 ± 0.1 mg/dl; − 2.81 ± 0.11 SDS, (n = 41), alkaline phosphatase (892 ± 84 mU/ml) (n = 39), 1,25(OH)_2_D62 ± 7 pg/ml (n = 34), parathyroid hormone (PTH)70 ± 7 pg/ml (n = 33), and tubular phosphate reabsorption (TPR) 69 ± 4% (n = 26).

**Table 3:** Biochemical manifestations at diagnosis

Patient	Serum phosphate		Serum alkaline phosphatases (mU/ml)	Serum 1,25(OH)_2_D (pg/ml)	Serum intact PTH (pg/ml)	TPR (%)
	mg/dl	SDS				
I.1	3.4	− 2.21	1230	87	–	–
I.2	3.8	− 1.05	1916	76	–	–
II.1	2.6	− 2.68	695	57	81	79
III.1	2.1	− 3.36	1646	16	68	32
IV.1	2.8	− 2.41	378	73	29	78
IV.2	3.0	− 2.44	232	40	43	82
V.1	2.1	− 3.99	1513	147	78	85
VI.1	2.6	− 2.68	525	33	64	78
VII.1	–	–	–	–	–	–
VII.2	2.4	− 2.95	516	–	–	–
VII.3	–	–	991	28	30	–
VII.4	–	–	–	–	–	–
VIII.1	3.1	− 2.27	598	55	39	38
IX.1	3.0	− 2.44	1824	47	59	67
X.1	2.9	− 2.61	639	33	136	74
XI.1	2.3	− 3.09	–	–	58	–
XII.1	2.7	− 2.54	–	–	48	–
XIII.1	–	–	–	–	–	–
XIV.1	3.3	− 2.34	892	64	65	75
XV.1	2.0	− 3.49	704	20	–	39
XVI.1	2.4	− 3.47	571	108	71	26
XVII.1	2.5	− 3.30	–	28	30	–
XVIII.1	2.3	− 3.09	1864	31	64	73
XIX.1	–	–	–	–	–	–
XX.1	2.9	− 2.27	446	88	116	93
XXI.1	3.1	− 2.27	470	56	49	86
XXII.1	2.8	− 2.78	697	74	32	76
XXIII.1	2.1	− 3.36	733	–	68	82
XXIV.1	2.2	− 3.22	514	61	78	58
XXV.1	2.9	− 2.27	1940	40	57	58
XXVI.1	2.3	− 3.64	423	–	–	82
XXVII.1	2.9	− 2.86	432	31	23	–
XXVII.2	–	–	–	–	–	–
XXVIII.1	3.0	− 2.14	236	78	54	–
XXVIII.2	3.3	− 2.34	426	171	57	90
XXIX.1	1.8	− 3.77	1555	–	–	–
XXX.1	3.1	− 2.00	829	183	101	88
XXX.2	2.2	− 3.22	158	25	–	–
XXXI.1	–	–	–	–	–	–
XXXII.1	2.8	− 2.41	692	41	111	–
XXXIII.1	2.1	− 3.99	–	–	76	54
XXXIV.1	2.3	− 3.09	856	64	101	46
XXXV.1	3.5	− 2.08	916	–	227	82
XXXV.2	2.2	− 3.22	1620	–	–	70
XXXVI.1	2.8	− 2.41	1093	65	66	–
XXXVII.1	2.4	− 2.95	1527	46	94	–
XXXVIII.1	2.4	− 2.95	856	40	58	–
XXXIX.1	3.0	− 2.14	759	51	43	–

1,25(OH)_2_D: 1,25 dihydroxyvitamin D. PTH: Parathyroid hormone

TPR: tubular phosphate reabsorption. Dash: information in this field was missing from the database

No differences were found between males and females for clinical manifestations, growth impairment or biochemical data at diagnosis. Likewise, no genotype–phenotype correlation was found. Actually, even patients within the same family presented different severity of clinical and biochemical manifestations.

**Fig. 3:**
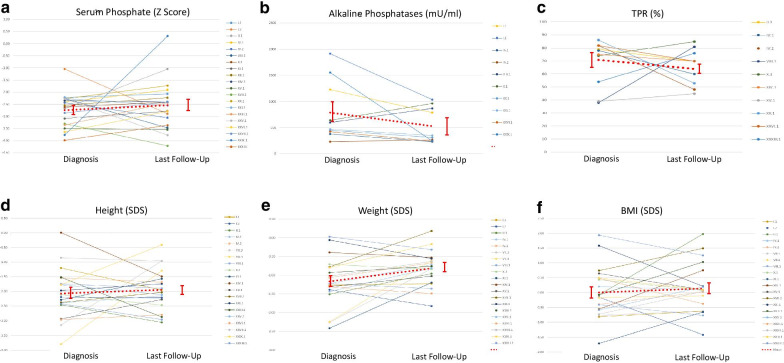
Biochemical and growth data at diagnosis and last follow-up. TPR: tubular phosphate reabsorption. BMI: Body Mass Index. Mean values are connected by red dots line. Vertical bars represent ± SEM

**Fig. 4:**
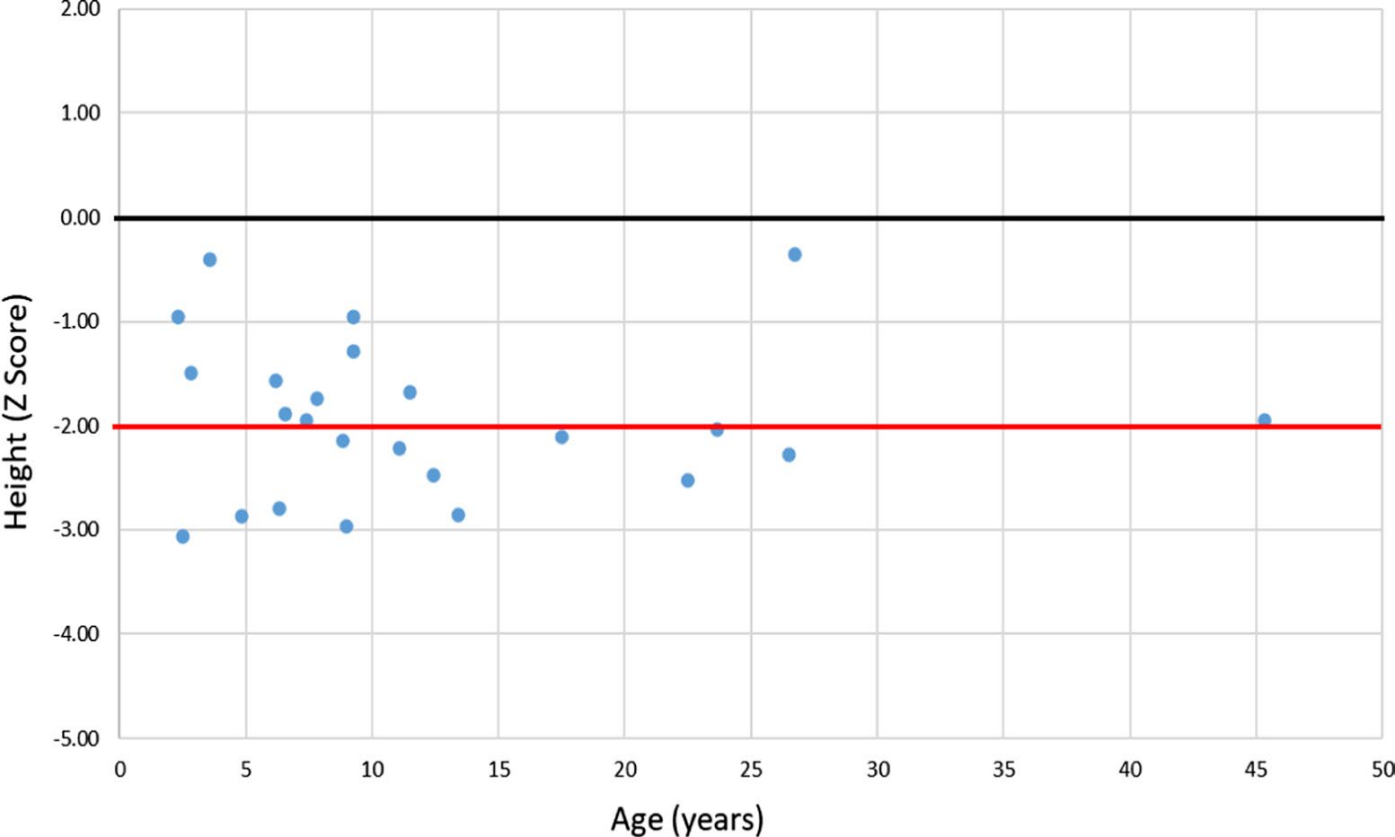
Height at last follow-up (n = 24). Black line: 0 SD; red line: − 2 SD

Growth and biochemical variables of 26 patients after a median follow uptime of 7.42 years (IQR = 11.26) are shown in Table 4, Figs. 3 and 4. Anthropometric data were − 1.94 ± 0.16 SDS for height (n = 24), − 0.82 ± 0.10 SDS for weight (n = 22) and 0.14 ± 0.19 SDS for BMI (n = 22). Comparison of data from patients with information both at diagnosis and last follow-up showed mean variations of 0.13 ± 0.23 SDS for height (p > 0.05) (n = 20), 0.35 ± 0.14 SDS for weight (p = 0.02) (n = 20) and 0.13 ± 0.20 SDS for BMI (p > 0.05) (n = 20).

**Table 4:** Biochemical manifestations at last follow up

Patient	Follow-up Time	Serum phosphate		Serum alkaline phosphatases (mU/ml)	Serum 1,25(OH)_2_D (pg/ml)	Serum intact PTH (pg/ml)	TPR (%)
		mg/dl	SD (Z score)				
I.1	7 y 2 m	2.9	− 2.07	787	–	30	72
I.2	10 y 1 m	2.7	− 2.90	1035	–	35	50
II.1	1 y 1 m	3.8	− 1.05	–	–	34	70
III.1	–	–	–	–	–	–	–
IV.1	7 y 6 m	3.0	− 1.91	231	78	32	60
IV.2	7 y 6 m	2.6	− 3.06	266	73	44	48
V.1	–	–	–	–	–	–	–
VI.1	–	–	–	–	–	–	–
VII.1	–	–	–	–	–	–	–
VII.2	–	–	–	–	–	–	–
VII.3	8 y 4 m	1.9	− 3.69	–	–	77	42
VII.4	8 y 3 m	1.8	− 3.85	665	–	108	73
VIII.1	2 y 7 m	2.4	− 3.47	868	15	84	81
IX.1	–	–	–	–	–	–	–
X.1	7 y 5 m	3.0	− 2.41	962	50	93	85
XI.1	5 y 11 m	2.8	− 2.78	632	45	49	42
XII.1	11 y 5 m	2.8	− 2.23	636	–	112	86
XIII.1	17 y 6 m	1.7	− 4.22	–	29	86	64
XIV.1	2 y 1 m	2.8	− 2.41	–	96	20	70
XV.1	25 y 5 m	2.0	− 3.55	–	20	75	45
XVI.1	–	–	–	–	–	–	–
XVII.1	18 y 8 m	1.7	− 4.22	120	32	99	42
XVIII.1	–	–	–	–	–	–	–
XIX.1	26 y 2 m	2.4	− 2.66	–	–	200	–
XX.1	1 y 9 m	3.3	− 1.73	312	72	53	45
XXI.1	3 y 5 m	2.6	− 2.56	348	43	26	53
XXII.1	–	–	–	–	–	–	–
XXIII.1	2 y 10 m	3.0	− 2.44	–	29	28	–
XXIV.1	–	–	–	–	–	–	–
XXV.1	16 y	1.9	− 3.77	–	–	37	–
XXVI.1	2 y 4 m	2.8	− 2.78	240	86	25	70
XXVII.1	1 y 10 m	2.8	− 2.41	–	58	49	76
XXVII.2	37 y 4 m	2.3	− 2.88	–	–	117	80
XXVIII.1	–	–	–	–	–	–	–
XXVIII.2	–	–	–	–	–	–	–
XXIX.1	1 y 10 m	4.8	0.31	244	68	73	89
XXX.1	–	–	–	–	–	–	–
XXX.2	–	–	–	–	–	–	–
XXXI.1	–	–	–	–	–	–	–
XXXII.1	–	–	–	–	–	–	–
XXXIII.1	4 y	2.1	− 3.36	–	–	114	76
XXXIV.1	–	–	–	–	–	–	–
XXXV.1	–	–	–	–	–	–	–
XXXV.2	–	–	–	–	–	–	–
XXXVI.1	–	–	–	–	–	–	–
XXXVII.1	–	–	–	–	–	–	–
XXXVIII.1	–	–	–	–	–	–	–
XXXIX.1	–	–	–	–	–	–	–

1,25(OH)_2_D: 1,25 dihydroxyvitamin D

m: month, y: year

PTH: parathyroid hormone

TPR: tubular phosphate reabsorption. Dash: information in this field was missing from the database

Mean SDS for serum phosphate was − 2.72 ± 0.20 (n = 25). Alkaline phosphatases, 1,25 (OH)_2_D and PTH levels were 525 ± 82 mU/ml (n = 14), 53 ± 7 pg/ml (n = 15) and 68 ± 8 pg/ml (n = 26) respectively. Mean tubular phosphate reabsorption was 65 ± 3% (n = 22). Comparison between diagnosis and last follow-up data revealed a variation of 0.20 ± 0.28 SDS for serum phosphate (p > 0.05) (n = 20), 5 ± 7 pg/ml for 1,25 (OH)_2_D (p > 0.05) (n = 10), 0 ± 11 pg/ml for PTH (p > 0.05) (n = 16) and − 7 ± 8 for tubular phosphate reabsorption (p > 0.05) (n = 11).

Eight out of 24 patients with renal ultrasounds at last follow-up presented nephrocalcinosis (Table 5).

**Table 5:** Treatment and clinical data at last follow-up

Patient	Phosphorus element dose (mg/kg/day)	Vitamin D dose (µg/day)	Nephrocalcinosis
I.1	38^a^	0.50^c^	No
I.2	40^a^	0.50^c^	No
II.1	86^a^	1.20^d^	Yes
III.1	–	–	–
IV.1	83^a^	0.60^d^	No
IV.2	62^a^	1.00^d^	No
V.1	–	–	–
VI.1	–	–	–
VII.1	–	–	No
VII.2	–	–	–
VII.3	55^b^	0.50^c^	Yes
VII.4	52^b^	0.50^c^	Yes
VIII.1	90^a^	1.50^d^	Yes
IX.1	–	–	–
X.1	40^a^	0.25^d^	No
XI.1	83^a^	0.50^c^	No
XII.1	44^b^	0.29^d^	No
XIII.1	41^b^	0.25^c^	Yes
XIV.1	52^a^	1.20^d^	–
XV.1	41^b^	1.00^c^	No
XVI.1	–	–	–
XVII.1	45^b^	0.25^c^	Yes
XVIII.1	–	–	–
XIX.1	27^b^	0.50^c^	Yes
XX.1	32^a^	0.50^c^	No
XXI.1	63^a^	1.50^d^	No
XXII.1	–	–	–
XXIII.1	65^a^	1.10^d^	No
XXIV.1	–	–	–
XXV.1	38^a^	1.40^c^	–
XXVI.1	58^b^	0.25^c^	No
XXVII.1	48^b^	0.60^d^	No
XXVII.2	–	–	–
XXVIII.1	–	–	–
XXVIII.2	–	–	–
XXIX.1	–	0.10^d^	No
XXX.1	–	–	–
XXX.2	–	–	Yes
XXXI.1	–	–	–
XXXII.1	–	–	–
XXXIII.1	49^b^	0.75^c^	No
XXXIV.1	–	–	–
XXXV.1	–	–	–
XXXV.2	–	–	–
XXXVI.1	–	–	–
XXXVII.1	–	–	–
XXXVIII.1	–	–	–
XXXIX.1	–	–	–

^a^Phosphate was administered as a solution

^b^Phosphate was administered as tablets

^c^Corresponds to 1,25 dihydroxy vitamin D

^d^Corresponds to 1 hydroxy vitamin D

## Discussion

This study provides a current description of the phenotypic characteristics of a large cohort of Caucasian pediatric patients with XLH genetically confirmed. The sample is a broad representation of the Spanish children with XLH, coming from several hospitals scattered through the country and provides data at diagnosis and after a median follow-up of 7.42 years. The study confirms that growth retardation, bone deformities and active lesions of rickets are the main presenting manifestations of the disease, within a wide spectrum of symptoms. No significant differences were found between males and females as for the severity of the disease. It is of interest that a broad spectrum of *PHEX* gene variants, all of them already described as pathogenic, was found and no mutation was specifically prevalent in Spanish population. All variants were classified as pathogenic according to the American College of Medical Genetics and Genomics consensus [19]. There was a high phenotypical variability even among family members harboring the same mutations, suggesting that other genes and environmental factors may affect the severity of XLH, as reported by other authors [20, 21].

In addition, the study shows that conventional treatment with phosphate supplements and vitamin D metabolites does not lead to persistent correction of hypophosphatemia or reduction of renal wasting of phosphate and does not modify the circulating levels of calcitriol. Unfortunately, this study does not provide information on circulating FGF23 levels, given its retrospective design. At the last follow-up visit, 11 out of 25 patients had serum PTH values mildly elevated. In XLH, development of hyperparathyroidism is thought to be related with the pharmacological administration of phosphate [22]. Eight patients developed nephrocalcinosis during the follow-up period, a finding linked to the administration of phosphate and vitamin D that usually does not result in subsequent clinical complications [16].

Though conventional treatment has been described to heal active signs of rickets and may improve bone deformities [23], this study confirms that it does not lead to catch-up growth. Mean height Z score of the group of patients remained low, − 1.89 at diagnosis versus − 1.94 at the last visit, although Fig. 3 indicates that the individual patients’ response varied from marked improvement to worsening of growth impairment. Two patients, VII.3 and XV.1, transiently received growth hormone treatment and their heights improved + 2.19and + 0.66 SDS, respectively. It is of note that 4 out of 16 patients had BMI greater than + 1.00 SDS at the last follow-up visit. This percentage of 25% corresponds to the normal distribution of reference population and it indicates that tendency to overweight and obesity was not found in the group of XLH patients here reported, unlike other series that have recently drawn attention to these complications likely related with the sedentary life and restricted mobility of these patients [11]. In this regard, a slight but significant increase in weight was found during the follow-up period in our series.

Our study presents methodological limitations inherent to the retrospective analysis and to the fact that patients’ information was extracted from a database in which some data were missing and cannot be recovered. It is also of note the lack of information on the degree of adherence to medication of each patient as well as the different monitoring protocols among the participating centers. However, it is an observational clinical study describing a large cohort of Spanish pediatric patients with genetically confirmed XLH and it provides current and interesting information on the clinical and biochemical features of the disease, at diagnosis and follow-up after conventional treatment. Our findings could be used as reference for further studies using burosumab treatment.

## Conclusions

This study confirms that growth retardation and rickets were the most prevalent clinical manifestations at diagnosis in a large series of Spanish pediatric patients with XLH confirmed by identification of pathogenic variants in the *PHEX* gene. Traditional treatment with phosphate supplements and calcitriol did not improve height or corrected hypophosphatemia and was associated with a risk of hyperparathyroidism and nephrocalcinosis. The severity of the disease was similar in males and females and no phenotype-genotype association was found.

## Patients and methods

The RenalTube database including 48 patients, 15 males and 33 females, with the diagnosis of XLH confirmed by defect-of-function mutations found in the *PHEX* gene was retrospectively reviewed to obtain demographic information and clinical and biochemical manifestations at diagnosis and at the last annual follow-up. Genetic information was confirmed and formatted according to Genome Reference Consortium Human Build 38 patch release 13 (GRCh38.p13) [24]. Variants were analyzed in silico and classified according to recommendations from the consensus of the American College of Medical Genetics and Genomics and the Association for Molecular Pathology [19] as pathogenic, likely pathogenic, benign or likely benign. Results for the age are presented as median and interquartile range (IQR). Other variables are presented for the group as mean (X) ± standard error of the mean (SEM). Z score (SDS) of anthropometric values was calculated using Spanish age and sex-matched reference values [25]. Patients with height ≤ 2 SDS were considered to have longitudinal growth retardation according to World Health Organization standards [26]. Reference values for biochemical parameters were obtained from the laboratory of the Hospital Universitario Central de Asturias (HUCA) [27].

All patients received treatment with phosphate supplementation (dose range of phosphorus element: 27–90 mg/kg/day at last follow-up) and vitamin D metabolites (dose range: 0.25–1.5 µg/day at last follow-up), according to the criteria and indications given by their physicians (Table 5). Two patients (VII.3 and XV.1) received growth hormone treatment. None of them received burosumab treatment.

Information in RenalTube database was downloaded and formatted to an Excel database. All fields but reasons for consultation and genetic information were multichoice or numeric format.

Chi squared test was used to analyze differences between sex for binary (Yes/No) fields (growth retardation, bone deformities). F-test was used to assess variance equality between sex for anthropometric and biochemical values. T-test was used to analyze differences between sex for anthropometric and biochemical values. Paired T-test for means was used to analyze differences between diagnosis and last follow-up for anthropometric and biochemical values. T-test for unequal variances was used to compare age at diagnosis for patients with and without family history of the disease.

Phenotype—genotype correlation was assessed by isolating the most severe phenotypes (lowest serum concentrations, most severe growth retardation, highest levels of alkaline phosphatases) and comparing genetic mutations in these patients looking for big deletions, SNPs with entirely different amino acids or nonsense mutations.

## Data Availability

The datasets used and/or analyzed during the current study are available from the corresponding author on reasonable request.
