# Correction to: Phenotypic characterization of X-linked hypophosphatemia in pediatric Spanish population

**DOI:** 10.1186/s13023-021-01786-5

**Published:** 2021-04-01

**Authors:** Enrique Rodríguez-Rubio, Helena Gil-Peña, Sara Chocron, Leire Madariaga, Francisco de la Cerda-Ojeda, Marta Fernández-Fernández, Carmen de Lucas-Collantes, Marta Gil, María Isabel Luis-Yanes, Inés Vergara, Juan David González-Rodríguez, Susana Ferrando, Montserrat Antón-Gamero, Marta Carrasco Hidalgo-Barquero, Angustias Fernández-Escribano, Mº Ángeles Fernández-Maseda, Laura Espinosa, Aniana Oliet, Antonio Vicente, Gema Ariceta, Fernando Santos, RenalTubeGroup

**Affiliations:** 1 Pediatric Research, Medicine Department, University of Oviedo, Oviedo, Spain; 2 AGC Pediatría, Hospital Universitario Central de Asturias, Oviedo, Spain; 3 Servicio de Nefrología Pediátrica, Hospital Vall D’Hebron, Universitat Autónoma de Barcelona, Barcelona, Spain; 4 Servicio Nefrología Pediátrica, IIS Biocruces-Bizkaia, Universidad del País Vasco UPV/EHU, Hospital Universitario Cruces, Barakaldo, Spain; 5 Unidad de Nefrología Pediátrica, Hospital Virgen del Rocío, Sevilla, Spain; 6 Servicio Pediatría, Complejo Asistencial Universitario de León, León, Spain; 7 Servicio Nefrología, Hospital Niño Jesús, Madrid, Spain; 8 Servicio Pediatría, Hospital Universitario de Santiago de Compostela, Santiago de Compostela, Spain; 9 Servicio Pediatría, Hospital Universitario Nuestra Señora de Candelaria, Santa Cruz de Tenerife, Spain; 10 Servicio Pediatría, Complexo Hospitalario Universitario A Coruña (CHUAC), A Coruña, Spain; 11 Unidad de Nefrología, Hospital General Universitario Santa Lucia, Cartagena, Spain; 12 Servicio de Pediatría, Hospital Clínico Universitario de Valencia, Valencia, Spain; 13 Unidad Nefrología Pediátrica, Hospital Universitario Reina Sofa, Córdoba, Spain; 14 Unidad de Nefrología Pediátrica, Hospital Universitario de Badajoz, Badajoz, Spain; 15 ServicioNefrología Infantil, Hospital Infantil Gregorio Marañón, Madrid, Spain; 16 Unidad de Nefrología Pediátrica, Hospital Virgen de La Salud, Toledo, Spain; 17 Servicio Nefrología infantile, Hospital Universitario Infantil La Paz, Madrid, Spain; 18 Servicio Nefrología, Hospital Severo Ochoa, Leganés, Spain; 19 Servicio Pediatría, Hospital Vega Baja, Orihuela, Spain

## Correction to: Orphanet J Rare Dis (2021) 16:104 https://doi.org/10.1186/s13023-021–01729-0

Following the publication of the original article [1] the authors requested to update their funding declarations as follows:

This research has been partially funded by Kyowa Kirin Farmacéutica S.L.U., project PI17/01745 from Instituto de Salud Carlos III, Acción Estratégica en Salud 2017–2020 and FEDER funds, Fondo de Investigaciones Sanitarias (FIS), Fundación Nutrición y Crecimiento (FUNDNYC), Instituto de Investigación Sanitaria del Principado de Asturias (ISPA) and Fundación para la Investigación y la Innovación Biosanitaria del Principado de Asturias (FINBA).

The Funding section has also been updated in the original article.
